# Drug-Eluting Stents Versus Conventional Endovascular Therapies in Symptomatic Infrapopliteal Peripheral Artery Disease: A Meta-analysis

**DOI:** 10.1016/j.jscai.2022.100024

**Published:** 2022-04-11

**Authors:** Khalid Changal, Mitra Patel, Pratyush Pavan Devarasetty, Rachel Royfman, Spiro Veria, Rohit Vyas, Mohammed Mhanna, Neha Patel, Azizullah Beran, Mark Burket, Rajesh Gupta

**Affiliations:** a Cardiovascular Medicine, University of Toledo College of Medicine and Health Sciences, Toledo, Ohio; b Department of Medicine, University of Toledo College of Medicine and Health Sciences, Toledo, Ohio; c University of Toledo College of Medicine and Health Sciences, Toledo, Ohio

**Keywords:** Drug-eluting stents, infrapopliteal PAD, peripheral artery disease, angioplasty, critical limb ischemia

## Abstract

**Background:**

Balloon angioplasty is the standard endovascular treatment for symptomatic infrapopliteal peripheral artery disease (PAD). However, recent trials have studied the effectiveness of drug-eluting stents (DES) for infrapopliteal PAD.

**Objective:**

This study investigated the use of DES compared with standard endovascular techniques for treatment of infrapopliteal artery disease.

**Methods:**

This is a comprehensive systematic review and meta-analysis of 9 recent randomized controlled trials. The primary clinical outcome assessed was primary patency. The secondary outcomes were target lesion revascularization (TLR), major limb amputation, and all-cause mortality.

**Results:**

A total of 945 patients met the inclusion criteria. Patients treated with DES were found to have increased primary patency than control at maximum follow-up (hazard ratio [HR] 2.17, 95% confidence interval [CI] 1.58-2.97, *P* < .0001, I^2^ = 62%). A similar result was seen in the subgroup of patients with critical limb ischemia (HR 2.58, 95% CI 1.49-4.49, *P* = .0008, I^2^ = 75%). DES were associated with significantly lower rates of TLR than control at maximum follow-up (HR 0.48, 95% CI 0.33-0.68, *P* < .0001; I^2^ = 11%). There was no statistical difference between DES versus control in rates of major limb amputation and mortality.

**Conclusions:**

DES have superior primary patency and TLR rates with no difference in amputation and all-cause mortality rates compared with conventional endovascular therapies in patients with infrapopliteal PAD.

## Highlights

• Drug-eluting stents (DESs) are superior to conventional endovascular treatment for infrapopliteal peripheral artery disease.
• DES use is associated with higher rates of primary patency and lower rates of target lesion revascularization.
• No differences in amputation rates or all-cause mortality were identified.

## Central Illustration

**Figure:**
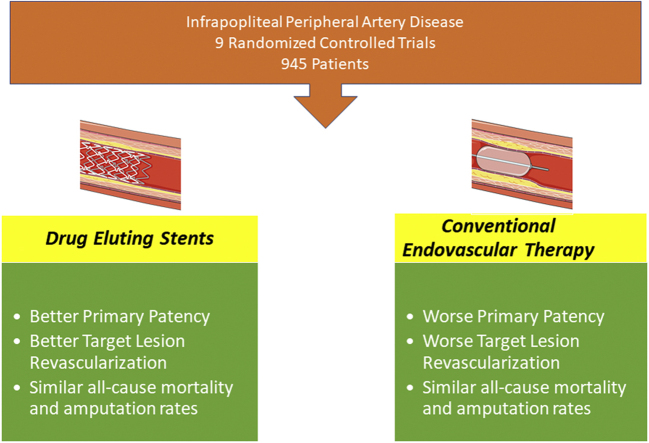

## Introduction

Balloon angioplasty with bailout stenting has been considered the standard endovascular therapy for many years for symptomatic infrapopliteal lesions.^1^^,^^2^ However, over the last 10 years, multiple small trials have shown the effectiveness of routine drug-eluting stents (DES) for infrapopliteal peripheral artery disease (PAD). DES have been associated with improved patency and lower rates of repeat revascularization.^1^, ^2^, ^3^ DES used in these studies were originally designed for coronary arteries; however, owing to the size similarities between coronary and below-the-knee arteries and superior efficacy of DES compared with bare metal stents (BMS) in the coronary arteries, DES have increasingly been used in infrapopliteal PAD in an off-label fashion.^4^

We performed a systematic review and meta-analysis of randomized controlled trials (RCTs) to investigate the use of DES compared with the current endovascular techniques that do not use DES for treatment of infrapopliteal artery disease.

## Methods

We performed a comprehensive search for applicable studies indexed in PubMed, EMBASE, and the Cochrane databases from inception to February 2021. A manual search was also performed for additional studies that met the inclusion criteria using the references of the included articles. The following search terms were used: “infrapopliteal disease” and “drug eluting stent.” Supplemental Table 1 describes the full search terms used.

**Figure 1:**
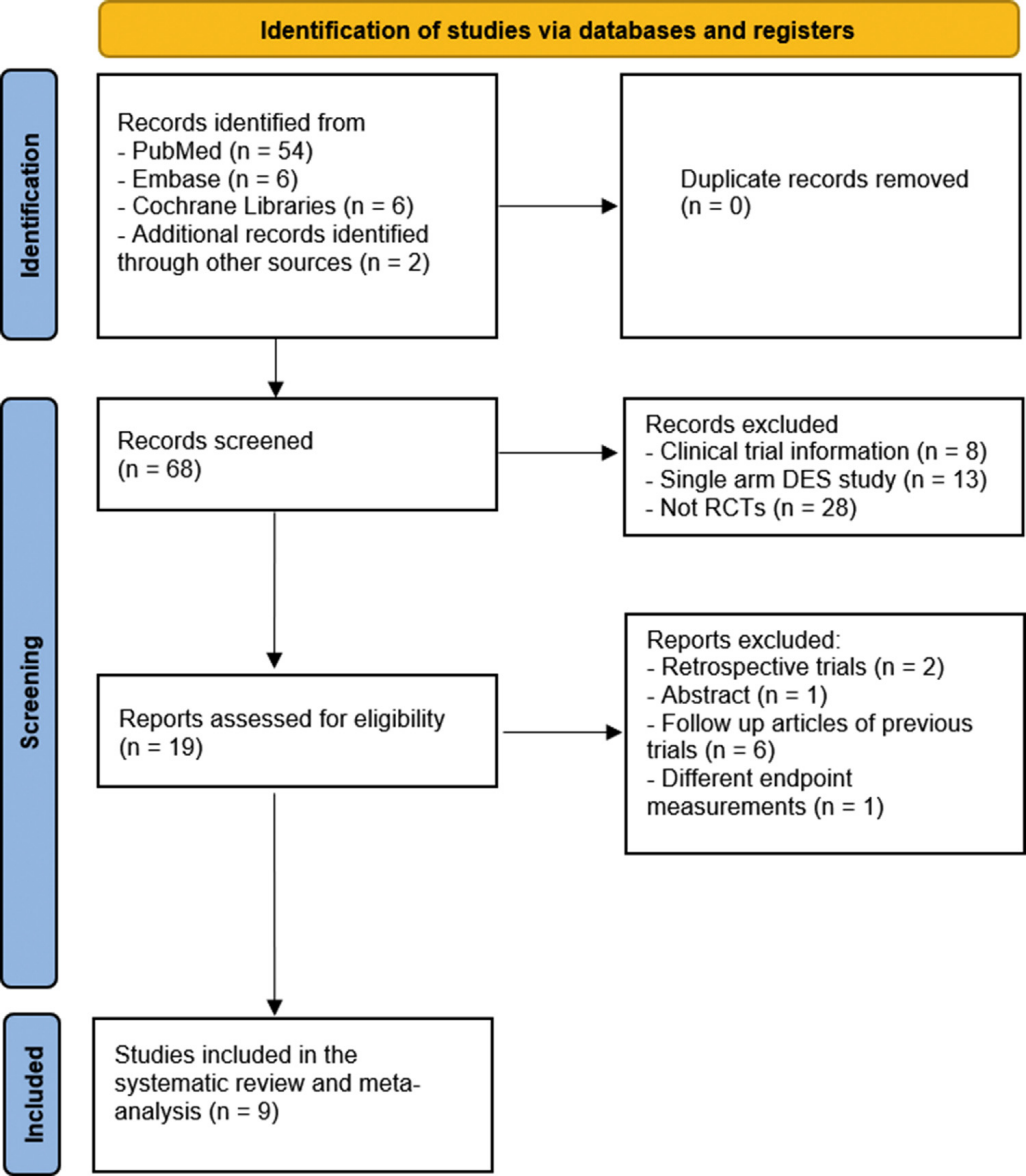
Identification of studies via databases and using Preferred Reporting Items for Systematic Reviews and Meta-analyses (PRISMA) guidelines.

The inclusion criteria for this meta-analysis were as follows: (1) adult patients (≥18 years of age) and (2) any full texts of controlled trials that evaluated the outcomes of DES versus percutaneous transluminal angioplasty/drug-coated balloons/BMS in adult patients with symptomatic infrapopliteal arterial occlusive disease with (3) follow-up of at least 6 months. Data extraction was independently performed by two investigators. Discrepancies were resolved by mutual discussion and from adjudications by a senior author. Demographic data and study characteristics were extracted. We followed the Preferred Reporting Items for Systematic Reviews and Meta-analyses statement guidelines to select the final studies (Fig. 1).^5^

The primary clinical outcome of our meta-analysis was primary patency. The secondary outcomes were target lesion revascularization (TLR), major limb amputation, and all-cause mortality. We performed a meta-analysis of the included studies using the Review Manager (RevMan) version 5.4.1 (The Cochrane Collaboration). The random-effects model was used to calculate the weighted hazard ratio (HR) and 95% confidence intervals (CIs) of our desired clinical outcomes. The HR was used as it adjusts for outcomes based on differences in follow-up. A *P*-value <.05 was considered statistically significant. Heterogeneity was assessed using the Higgins I^2^ index, where I^2^ values > 50% implied the presence of substantial heterogeneity. Meta regression was not performed as the number of studies used for each outcome was less than 10. We assessed the quality of the included studies using the revised Cochrane risk-of-bias tool for randomized trials (RoB 2) for RCTs. Two authors independently assessed each study for bias.

## Results

A total of 9 studies, which included a total 945 patients, met our inclusion criteria and were included in the meta-analysis. Table 1 shows the baseline characteristics of the studies included in the meta-analysis. All studies were published since 2007. Most of the studies were conducted in Europe. The mean age range of the patients in the studies was 68.8 to 75 years with the majority being male (58%-79% of study participants). Diabetes mellitus prevalence ranged from 54% to 82.5%, hypertension from 50% to 90.5%, hyperlipidemia from 34.9% to 76.6%, and smoking from 6.3% to 44%. The follow-up periods ranged from 6 months to 48 months among the 9 studies. Table 2 further characterizes the inclusion criteria along with the specific intervention control group.

**Table 1:** Baseline characteristics of the patients included in the meta-analysis.

Study	DES, n	Control, n	Mean age, yrs	Men, %	HTN, %	HLD, %	DM, %	Smokers, %	Lesion length, mm	Vessel diameter, mm	Longest follow-up period, mo	DAPT duration, mo
Siabilis et al (2007)^6^	29	29	68.8	79	85.4	68.9	82.5	41.7	54.9	<4	36	6
Falkowski et al (2009)^7^	25	25	69.4	58	62	36	66	44	17.8	2.7	6	6
Tepe et al (BELOW 2010)^8^	14	49	72.4	64	68.2	34.9	68	6.3	34	2.9	36	2
Rastan et al (YUKON BTK 2011)^9^	82	79	73	67	68.6	37.9	54	28.5	31	3	33	6
Karnabatidis et al (2011)^10^	47	34	71	78	85	64	75.3	40	76	–	36	6
Bosiers et al (DESTINY 2012)^11^	74	66	75	64	89.9	76.6	55	13	15.9	3	12	12
Scheinert et al (ACHILLES 2012)^12^	99	101	73.4	72	90.5	72.95	64.5	32.3	26.9	2.6	36	6
Siabilis et al (IDEAS 2014)^13^	27	25	71.5	76	50	47	70	30	138	–	6	6
Spreen et al (PADI 2016)^14^	66	74	73.6	70	–	–	63.5	24.1	22	2.9	48	At least 6

DAPT, dual antiplatelet therapy; DES, drug-eluting stent; DM, diabetes mellitus; HLD, hyperlipidemia; HTN, hypertension.

**Table 2:** Primary endpoints, inclusion criteria, and exclusion criteria of the studies included in the meta-analysis.

Study	DES drug	Control	Primary endpoint	Secondary endpoints	Inclusion criteria	Exclusion criteria
Siabilis et al (2007)^6^	Sirolimus	BMS	Primary patency	Binary in-stent restenosis, binary in-segment restenosis, TLR, all-cause mortality, limb salvage, and major and minor amputations	Rutherford class 4-6 with critical limb ischemia symptoms, target vessel diameter less than 4 mm, DSA documentation of infrapopliteal obstructive arterial disease, bailout stenting after suboptimal and/or complicated below-the-knee angioplasty	Buerger's disease, allergy to contrast, aspirin, or antiplatelets, coagulation disorders, acute limb ischemia, deep vein thrombosis, infected tissue loss, target vessel aneurysms, bilateral critical limb ischemia
Falkowski et al (2009)^7^	Sirolimus	BMS	Restenosis	Minimum luminal diameter, late lumen loss, diameter stenoses, target lesion revascularization, technical success, clinical patency, ABI.	Greater than 30 years old, PAD Rutherford class 3-5, primary stenosis greater 60%, target vessel length 0.5-3 cm, target vessel diameter 2-3.5 mm	Buerger's disease, target vessel aneurysm, previous percutaneous treatment within the target vessel, presence of peripheral vascular prosthesis, severe calcification or tortuosity of the vessel, thrombophlebitis, severe renal insufficiency, liver failure, immunosuppressant therapy, recent hemorrhagic stroke (3 mo), allergy to aspirin, clopidogrel, heparin or contrast, active infection, life expectancy less than 2 years
Tepe et al (BELOW 2010)^8^	Sirolimus	BMS/PTA	Restenosis	Clinical patency, ulceration healing, major amputation, all-cause mortality, technical success.	Greater than 18 years old, PAD Rutherford class 5-6, target vessel diameter less than 5 cm	Target vessel aneurysm, acute limb ischemia, active bleeding, medication-induced thrombocytopenia, major surgery, eye surgery or trauma within 6 weeks of the procedure, life expectancy less than 1 year
Karnabatidis et al (2011)^10^	Everolimus	BMS	Primary patency Restenosis	Patient survival, major amputation-free survival, angiographic, overall event-free survival.	Rutherford class 4-6, identification of at least 1 hemodynamically significant (≥70% by visual estimation) infrapopliteal stenosis or occlusion ≥4.5 cm long documented by either digital subtraction angiography (DSA) or computed tomographic angiography	Lifestyle limiting intermittent claudication, acute limb ischemia, Buerger disease, deep vein thrombosis, infected tissue loss, a history of severe contrast allergy or hypersensitivity, intolerance to aspirin and/or clopidogrel, systemic coagulopathy, or hypercoagulation disorders.
Rastan et al (YUKON BTK 2011)^9^	Sirolimus	BMS	Primary patency	Secondary patency, ABI, Rutherford class	Greater than 21 years old, PAD Rutherford class 2-5, primary stenosis of greater than 70%, target vessel length less than 45 mm, target vessel diameter 2.3-3.5 mm	Buerger's disease, visible thrombus within the target lesion, acute limb ischemia, coagulation disorder, known allergy to contrast, heparin, or antiplatelet therapy, life expectancy less than 1 year.
Bosiers et al (DESTINY 2012)^11^	Everolimus	BMS	Primary patency	Freedom from TLR, Rutherford class, all-cause mortality, late lumen loss, diameter stenosis, limb salvage.	Greater than 18 years old, PAD Rutherford 4-5 with symptomatic critical limb ischemia, less than or equal to 2 focal target lesions in the infrapopliteal vessels, target vessel diameter 2-3.5 mm, target vessel length <4 cm	Previous percutaneous treatment within the target vessel, presence of peripheral vascular prosthesis, lesion requiring kissing stent procedure, target vessel aneurysm, allergy to heparin, aspirin, antiplatelet therapies, or other anticoagulants, coagulation disorder, use of alternative therapy for peripheral vascular disease (eg, laser, radiation, cutting balloon, etc.), greater than 2 infrapopliteal lesions in the same limb
Scheinert et al (ACHILLES 2012)^12^	Sirolimus	PTA	Restenosis	Diameter stenosis, minimum luminal diameter, late lumen loss, Doppler-detected patency, TLR, stent fracture, procedural success, device success, lesions success, periprocedural complications, serious adverse events, Rutherford class, ABI, major amputation, quality of life assessment	Greater than 18 years old, PAD Rutherford 3-5 with symptomatic critical limb ischemia, primary stenosis greater than 70%, target vessel diameter 2.5-3.5 mm	Active infection, renal insufficiency, allergies or contraindication to aspirin, antiplatelet therapies, heparin, stainless steel, or contrast agent, warfarin use, any medical comorbidities that interfere with the subjects' participation in the study per investigators opinion, life expectancy less than 1 year.
Siabilis et al (IDEAS 2014)^13^	Zotarolimus, sirolimus, everolimus	DCB (paclitaxel)	Restenosis	Success rate, periprocedural complications, TLR, limb salvage rates, primary patency	Greater than 18 years old, PAD Rutherford class 3-6, lesion length 70-220 mm	Lesion length less than 70 mm or greater than 220 mm, lesions in the distal 3rd of the tibial vessels, patients without distal runoff
Spreen et al (PADI 2016)^14^	Paclitaxel	PTA/BMS	Primary Patency	Major amputation, minor amputation, infrapopliteal surgical bypass, TLR, periprocedural complications, all-cause mortality	Greater than 18 years old, PAD Rutherford class 4-6 with critical limb ischemia, primary stenosis greater than 50%, lesion length less than 6 cm, target vessel diameter 2-4 mm	Acute or subacute limb ischemia requiring thrombolysis, recent hemorrhagic stroke or GI/GU bleeding within the past 3 months, vessel aneurysm, previous percutaneous treatment within the target vessel, known allergy to aspirin, clopidogrel, heparin, paclitaxel, contrast media, heparin-induced thrombocytopenia, renal insufficiency (not counting dialysis patients), severely calcified lesions, poor vessel inflow, significant vessel tortuosity, patient without distal runoff, life expectancy less than 6 months

ABI, ankle-brachial index; BMS, bare metal stents; DCB, drug-coated balloon; PAD, peripheral artery disease; PTA, percutaneous transluminal angioplasty; TLR, target lesion revascularization.

**Figure 2:**
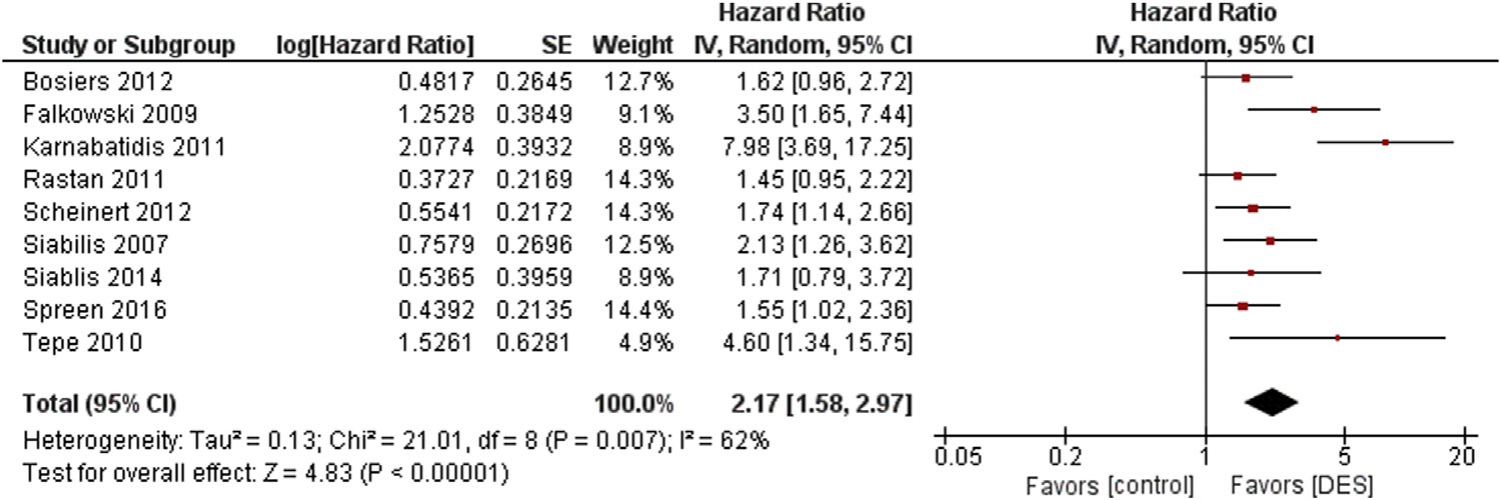
Primary patency results for DES versus control. DES, drug-eluting stent.

The primary clinical outcome was primary patency. The patients treated with DES showed a significantly increased primary patency in infrapopliteal disease compared with the control group at maximum follow-up (HR 2.17, 95% CI 1.58-2.97, *P* < .0001, I^2^ = 62%) as seen in Figure 2. A similar result was seen in the subgroup of patients with critical limb ischemia (CLI) (HR 2.58, 95% CI 1.49-4.49, *P* = .0008, I^2^ = 75%) (Supplemental Figure 1). Invasive angiography was used for assessment of primary patency in 7 of 9 studies. Falkowski et al^7^ used duplex ultrasound, whereas Spreen et al^14^ used computed tomographic angiography.

**Figure 3:**
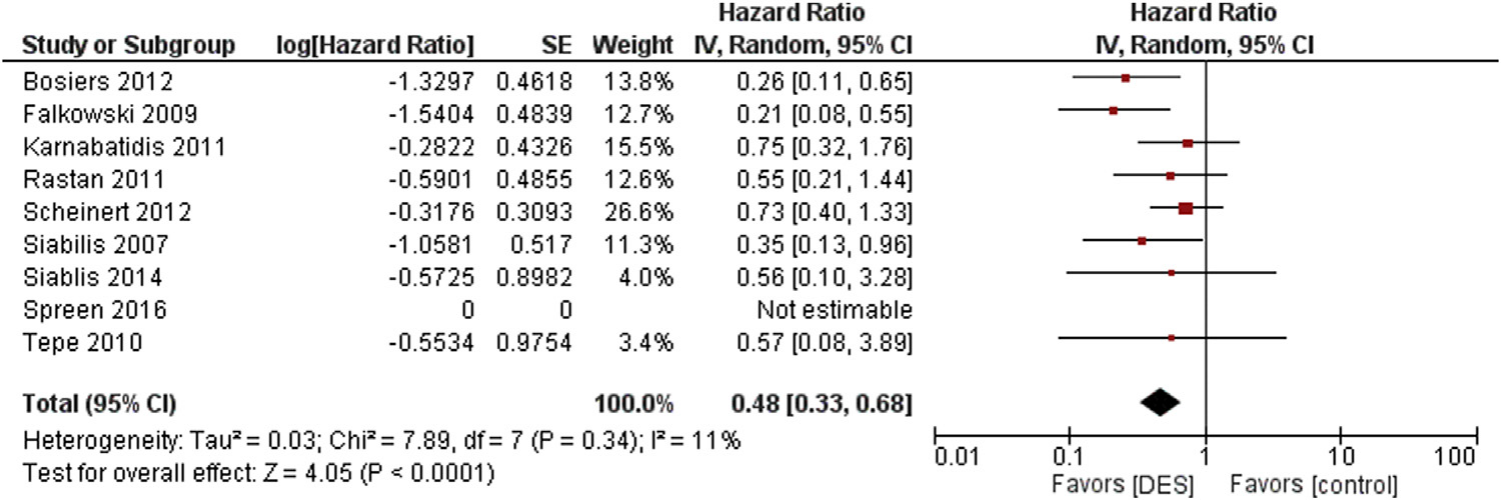
Target lesion revascularization outcomes for DES versus control. DES, drug-eluting stent.

The secondary clinical outcomes were TLR, major limb amputation, and all-cause mortality. Compared with control, DES were significantly associated with a decreased TLR (HR 0.48, 95% CI 0.33-0.68, *P* < .0001; I^2^ = 11%) as seen in Figure 3. In patients with critical limb ischemia, the DES was shown to be associated with a decreased rate of TLR as compared with control (HR 0.44, 95% CI 0.26-0.73, *P* = .002, I^2^ = 2%) (Supplemental Figure 2).

**Figure 4:**
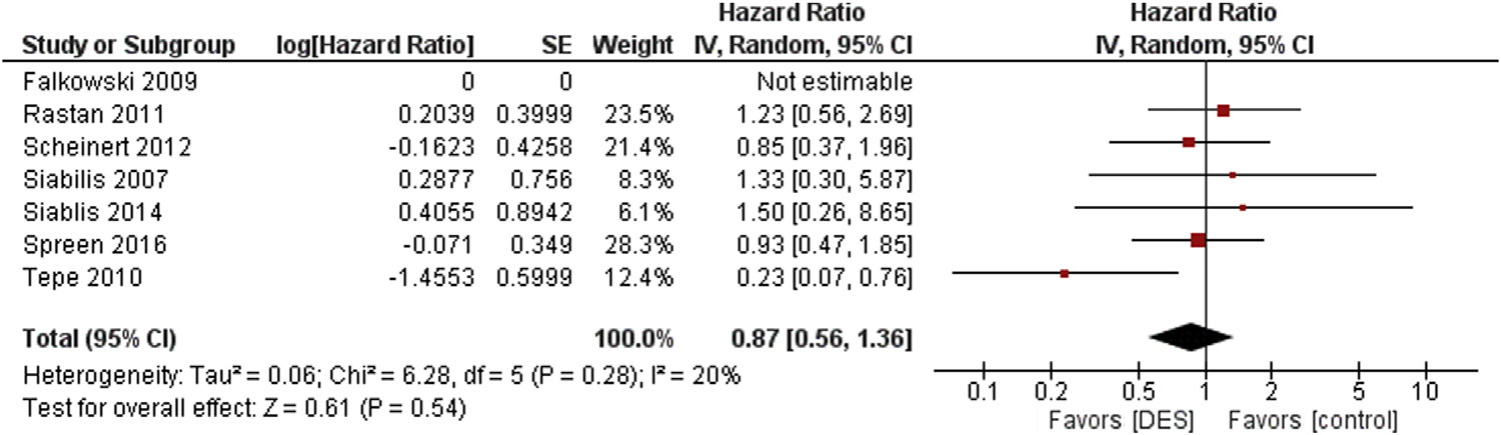
Major amputation occurrence among DES versus control. DES, drug-eluting stent.

There was no statistical difference between DES versus control for subsequent major amputations seen at maximum follow-up (HR 0.87, 95% CI 0.56-1.36, *P* = .28, I^2^ = 20%) (Fig. 4). Similar results were seen in patients with CLI (HR 0.64, 95% CI 0.40-1.04, *P* = .07, I^2^ = 0%) (Supplemental Figure 3).

**Figure 5:**
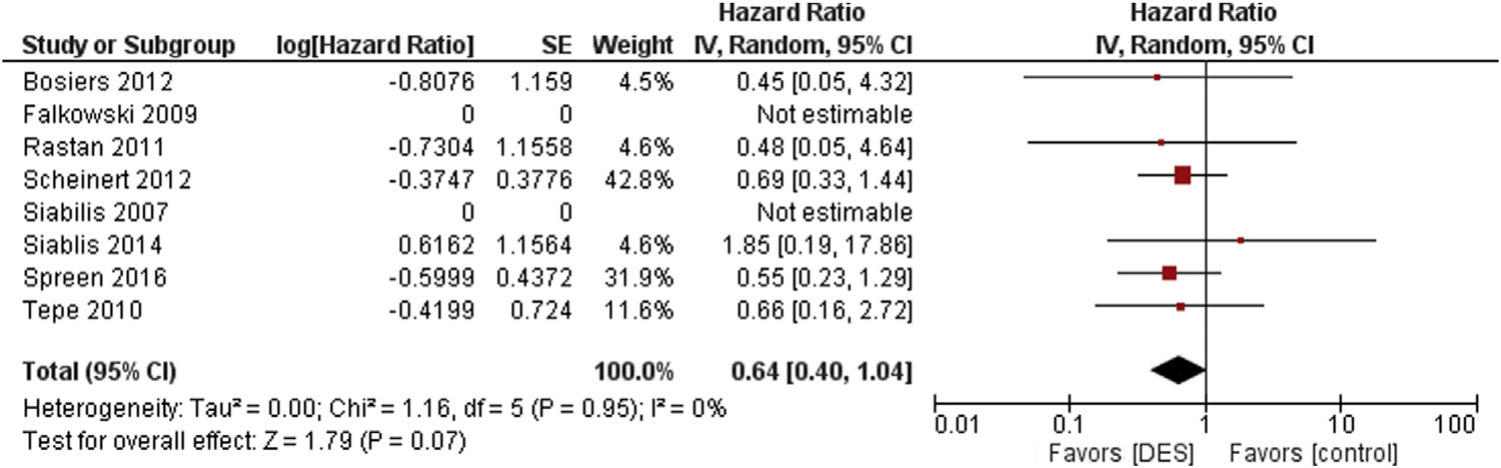
All-cause mortality rates among DES versus control. DES, drug-eluting stent.

There was also no statistically significant difference in all-cause mortality in patients with infrapopliteal disease who received the DES versus control at maximum follow-up (HR 0.64, 95% CI 0.40-1.04, *P* = .95, I^2^ = 0%] (Fig. 5). In patients with CLI, there was no statistically significant difference in all-cause mortality between the DES versus control (HR 0.66, 95% CI 0.25-1.73, *P* = .40, I^2^ = 58%) (Supplemental Figure 4).

**Central Illustration:**
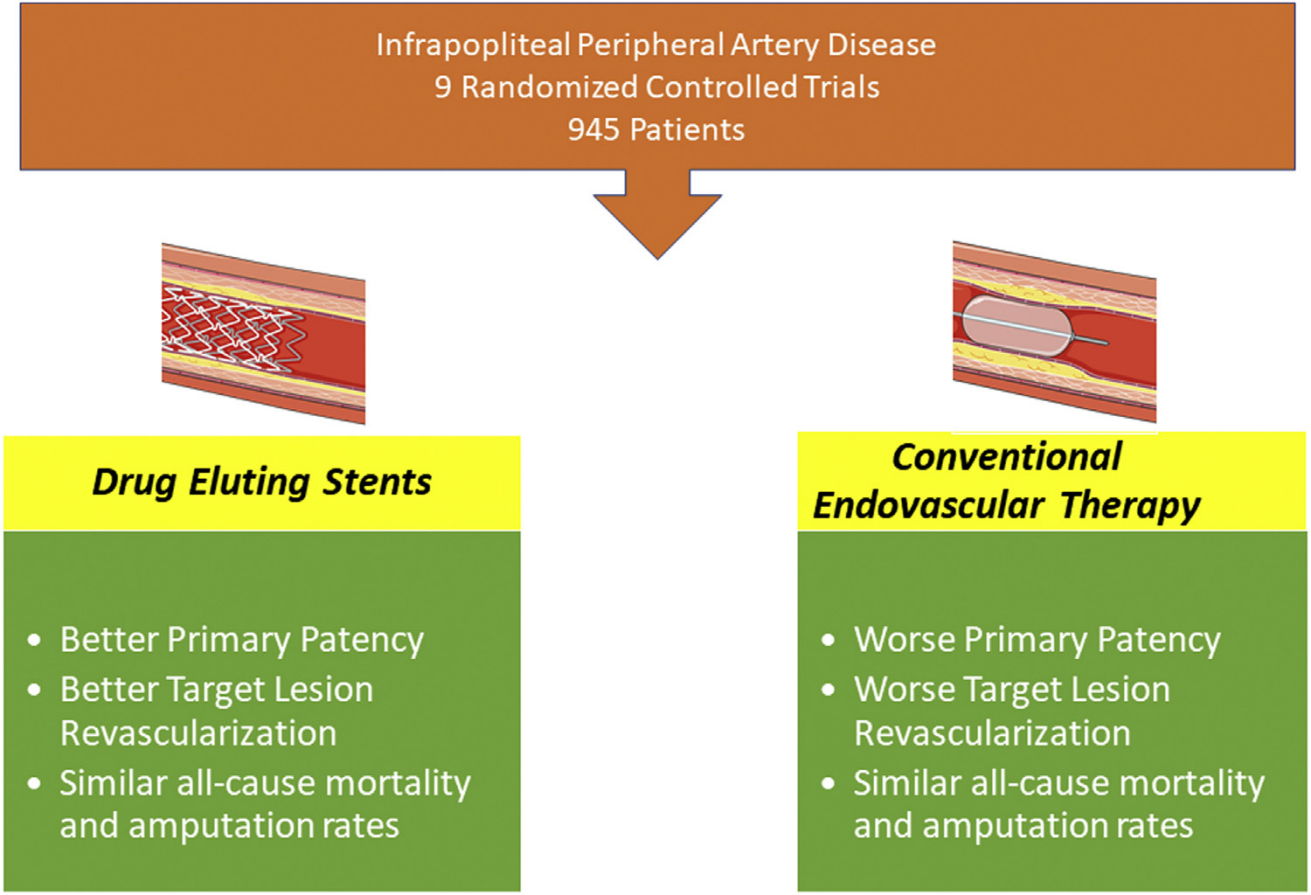
Drug-eluting stents are superior to conventional endovascular therapies for symptomatic infrapopliteal peripheral artery disease.

The revised Cochrane risk-of-bias tool for randomized trials (RoB 2) for RCTs was used to assess bias. The studies by Bosiers,^11^ Rastan,^9^ and Scheinert^12^ had a low overall risk of bias. The studies by Falkowski,^7^ Karnabatidis,^10^ Siablis (2007),^6^ Siablis (2014),^13^ Spreen,^14^ and Tepe^8^ had some concerns of bias. Karnabatidis^10^ was limited by the randomization process of the study design as the control group was chosen retrospectively, whereas Siablis (2007)^6^ raised some concerns of bias as there was no randomization sequence specified. The other 4 studies had study designs where the assessment of the outcome could have been influenced by the knowledge of intervention received, which led to some concerns of risk of bias.

## Discussion

Our meta-analysis is the most comprehensive study consolidating randomized control trials that primarily evaluated the use of DES for infrapopliteal PAD. This study includes 9 multicenter RCTs enrolling 945 patients (463 in the DES and 482 in the control group) with the majority following the patients for 12 months or longer.^6^, ^7^, ^8^, ^9^, ^10^, ^11^, ^12^, ^13^, ^14^ The primary purpose of this systematic review and meta-analysis was to provide the most up-to-date evidence based on prior RCTs in the efficacy and safety of DES versus other modes of endovascular therapies for infrapopliteal PAD. The major results of the study are that DES have better primary patency and decreased TLR than other forms of endovascular revascularization therapies. We did not see any evidence of increased mortality or major amputation rates in patients treated with DES (Central Illustration).

In the past, stents were used only as a bailout procedure during endovascular treatment of infrapopliteal disease.^15^, ^16^ However, the similarity between the size of infrapopliteal and coronary arteries stimulated the investigation of the DES as a primary treatment strategy for infrapopliteal artery disease.^10^, ^11^, ^12^ DES have shown superiority over bare metal stents and balloon angioplasty in coronary arteries.^17^ We reviewed the randomized control trials that have tested the use of DES in infrapopliteal PAD in this study. Although randomized control trials have consistently shown improved efficacy and safety with DES, these are limited by smaller sample size.^6^, ^7^, ^8^, ^9^, ^10^, ^11^, ^12^, ^13^, ^14^ Our meta-analysis pools together the data from all the randomized control trials and is the largest on this topic so far. Although there have been prior meta-analyses on this topic, these are limited by older studies and small or variable follow-up.^18^, ^19^, ^20^ In our meta-analysis, we use the HR as an outcome to measure the efficacy of DES. The HR adjusts for the variable follow-up that is often encountered on pooling of data from different RCTs and, thus, is a better measure of outcomes.^21^^,^^22^ This methodological difference could explain the conflicting results at 1- and 3-year follow-up seen in a prior meta-analysis.^19^ In addition, prior meta-analyses have been limited to studies conducted on lesions that were only short.^18^ However, in clinical practice, the infrapopliteal lesions are often long. Our study analyzes studies with both short and long lesions and, thus, overcomes this limitation encountered in prior meta-analyses. In addition, we have performed subgroup analysis on patients with critical limb ischemia and found similar results of improved primary patency and lower TLR rates with DES. The most comprehensive prior meta-analysis on this subject was limited by certain statistical shortcomings such as utilization of a fixed effect rather than a random-effects model for measurement of effect size.^20^ A random-effects model is appropriate to adjust for the heterogeneity of the included studies and was utilized in the present study. In addition, the odds ratio was used to calculate pooled effect size in the prior study. The follow-up of the studies included ranged from 6 to 48 months. The odds ratio may provide less accurate results with such a variable follow-up. The HR, as used in the present study, is a more accurate marker of effect size.^21^ In addition to addressing the above mentioned shortcomings, we provide a more detailed and updated analysis of RCTs.

The current iteration in the design of coronary DES provides unique benefits for treating infrapopliteal arterial lesions. These benefits include thin strut design, lower thrombogenicity, and easy deliverability. Balloon angioplasty of infrapopliteal lesions is often limited by elastic recoil in the short term and restenosis in the medium-to-long term.^23^ Bare metal stents used in infrapopliteal arteries are also limited by restenosis. DES provide strong radial force to counter recoil, and the local delivery of antiproliferative drugs prevents neointimal proliferation and restenosis.^24^, ^25^, ^26^, ^27^, ^28^

Although our study showed improved primary patency and TLR rates with DES, this did not translate into an all-cause mortality benefit. The predominant cause for mortality in patients with PAD is cardiac disease and treatment of infrapopliteal arterial disease with endovascular interventions would not be expected to reduce cardiac mortality.^28^ Nonetheless, although mortality was not significantly different, improvements in limb-specific outcomes such as primary patency and TLR are clinically meaningful.

DES is the standard of care for percutaneous treatment of severe coronary artery disease and is gaining pace in infrapopliteal endovascular interventions as well.^29^ Our study provides comprehensive evidence that DES should be strongly considered in infrapopliteal peripheral arteries. In light of this analysis, it is time for a paradigm shift with consideration of DES as a primary treatment option for infrapopliteal artery disease in appropriate patients rather than a bailout strategy.

Newer technologies such as atherectomy, Tack Endovascular System, and bioresorbable vascular scaffolds are also emerging as effective invasive treatment strategies for infrapopliteal artery disease.^30^, ^31^, ^32^ Additional studies comparing DES with these other techniques/technologies are required.

There are several limitations to our meta-analysis. There is some variation of inclusion criteria between the studies. As seen in Table 2, definition of certain endpoints varied between some studies. Primary patency was assessed by invasive angiography in 7 of 9 studies, noninvasively by computed tomography angiography in one, and duplex ultrasound in one study. In addition, the DES group used different active drugs such as sirolimus, everolimus, and paclitaxel, each of which may be associated with different efficacy. The control group is heterogenous with variable frequency of the use of BMS in different studies, and one study used a drug-coated balloon^13^ in the control group. The control group does not accurately reflect the contemporary practice of endovascular treatment of infrapopliteal PAD but is the closest reflection of current practice within the available literature. We included studies with both short and long infrapopliteal lesions. In clinical practice, infrapopliteal lesions are often very long. Current therapy has adapted by typically using very long (eg, 150-200 mm) balloons. There is no comparable length DES and there are practical limitations to DES use in the distal segments of the infrapopliteal arteries such as external compression. For very long lesions, a practical approach may be long balloon percutaneous transluminal angioplasty followed by limited DES in the proximal segments of the infrapopliteal arteries. Alternative methods that minimize the metal implants in these very long lesions (eg, Tack Endovascular System)^30^ were not studied in this analysis. The use of atherectomy for infrapopliteal arterial disease was not studied in this meta-analysis.

## Conclusion

In conclusion, this meta-analysis has demonstrated that DES have superior primary patency and TLR rates with no difference in amputation and all-cause mortality rates compared with conventional endovascular therapies in patients with infrapopliteal PAD.
