# Supplementary material for: Drug-Eluting Stents Versus Conventional Endovascular Therapies in Symptomatic Infrapopliteal Peripheral Artery Disease: A Meta-analysis
Source: J Soc Cardiovasc Angiogr Interv. 2022 Apr 11;1(2):100024. doi: 10.1016/j.jscai.2022.100024 (PMC11308017; doi:10.1016/j.jscai.2022.100024)
Supplement: Supplementary File [file mmc1.docx]

**Supplementary Material**

**PUBMED SEARCH STRATEGY 10/25/2019**

("Infrapopliteal disease" OR "below the knee" OR "infrapopliteal artery" OR "angioplasty" OR "drug-eluting stent(s)" OR "bare metal stent(s)" OR “drug-coated ballon(s)” OR "trial" OR “randomized trial” OR “retrospective” OR “critical limb ischemia” OR “bail out stenting” OR “primary patency” OR “target lesion reintervention”)

Total (filtered for “clinical trials”)=54 articles.

**EMBASE SEARCH STRATEGY 10/25/2019**

("Infrapopliteal disease" OR "below the knee" OR "infrapopliteal artery" OR "angioplasty" OR "drug-eluting stent(s)" OR "bare metal stent(s)" OR “drug-coated ballon(s)” OR "trial" OR “randomized trial” OR “retrospective” OR “critical limb ischemia” OR “bail out stenting” OR “primary patency” OR “target lesion reintervention”)

Total (filtered for “clinical trials”)=6 articles

**COCHRANE LIBRARIES SEARCH STRATEGY 10/25/2019**

("Infrapopliteal disease" OR "below the knee" OR "infrapopliteal artery" OR "angioplasty" OR "drug-eluting stent(s)" OR "bare metal stent(s)" OR “drug-coated ballon(s)” OR "trial" OR “randomized trial” OR “retrospective” OR “critical limb ischemia” OR “bail out stenting” OR “primary patency” OR “target lesion reintervention”)

Total (filtered for “clinical trials”)=6 articles

**Supplementary Table 1:** **PRISMA Checklist**

| **Section/topic** | **#** | **Checklist item** | **Reported on page #** |
| --- | --- | --- | --- |
| **TITLE** | | |  |
| Title | 1 | Identify the report as a systematic review, meta-analysis, or both. | 1 |
| **ABSTRACT** | | |  |
| Structured summary | 2 | Provide a structured summary including, as applicable: background; objectives; data sources; study eligibility criteria, participants, and interventions; study appraisal and synthesis methods; results; limitations; conclusions and implications of key findings; systematic review registration number. | 2 |
| **INTRODUCTION** | | |  |
| Rationale | 3 | Describe the rationale for the review in the context of what is already known. | 3 |
| Objectives | 4 | Provide an explicit statement of questions being addressed with reference to participants, interventions, comparisons, outcomes, and study design (PICOS). | 3,4 |
| **METHODS** | | |  |
| Protocol and registration | 5 | Indicate if a review protocol exists, if and where it can be accessed (e.g., Web address), and, if available, provide registration information including registration number. | NA |
| Eligibility criteria | 6 | Specify study characteristics (e.g., PICOS, length of follow-up) and report characteristics (e.g., years considered, language, publication status) used as criteria for eligibility, giving rationale. | 4 |
| Information sources | 7 | Describe all information sources (e.g., databases with dates of coverage, contact with study authors to identify additional studies) in the search and date last searched. | 4 |
| Search | 8 | Present full electronic search strategy for at least one database, including any limits used, such that it could be repeated. | Supplementary page 2 |
| Study selection | 9 | State the process for selecting studies (i.e., screening, eligibility, included in systematic review, and, if applicable, included in the meta-analysis). | Figure 1 |
| Data collection process | 10 | Describe method of data extraction from reports (e.g., piloted forms, independently, in duplicate) and any processes for obtaining and confirming data from investigators. | 3,4 |
| Data items | 11 | List and define all variables for which data were sought (e.g., PICOS, funding sources) and any assumptions and simplifications made. | 4 |
| Risk of bias in individual studies | 12 | Describe methods used for assessing risk of bias of individual studies (including specification of whether this was done at the study or outcome level), and how this information is to be used in any data synthesis. | 4,5 |
| Summary measures | 13 | State the principal summary measures (e.g., risk ratio, difference in means). | 4 |
| Synthesis of results | 14 | Describe the methods of handling data and combining results of studies, if done, including measures of consistency (e.g., I^2^) for each meta-analysis. | 3,4,5 |

| **Section/topic** | **#** | **Checklist item** | **Reported on page #** |
| --- | --- | --- | --- |
| Risk of bias across studies | 15 | Specify any assessment of risk of bias that may affect the cumulative evidence (e.g., publication bias, selective reporting within studies). | 6,7 |
| Additional analyses | 16 | Describe methods of additional analyses (e.g., sensitivity or subgroup analyses, meta-regression), if done, indicating which were pre-specified. | 6,7 |
| **RESULTS** | | |  |
| Study selection | 17 | Give numbers of studies screened, assessed for eligibility, and included in the review, with reasons for exclusions at each stage, ideally with a flow diagram. | Figure 1 |
| Study characteristics | 18 | For each study, present characteristics for which data were extracted (e.g., study size, PICOS, follow-up period) and provide the citations. | Table 2 |
| Risk of bias within studies | 19 | Present data on risk of bias of each study and, if available, any outcome level assessment (see item 12). | Suppl 10-22 |
| Results of individual studies | 20 | For all outcomes considered (benefits or harms), present, for each study: (a) simple summary data for each intervention group (b) effect estimates and confidence intervals, ideally with a forest plot. | Figure 2-5 |
| Synthesis of results | 21 | Present results of each meta-analysis done, including confidence intervals and measures of consistency. | Figure 2-5 |
| Risk of bias across studies | 22 | Present results of any assessment of risk of bias across studies (see Item 15). | 8 |
| Additional analysis | 23 | Give results of additional analyses, if done (e.g., sensitivity or subgroup analyses, meta-regression [see Item 16]). | 9-15 |
| **DISCUSSION** | | |  |
| Summary of evidence | 24 | Summarize the main findings including the strength of evidence for each main outcome; consider their relevance to key groups (e.g., healthcare providers, users, and policy makers). | 5-6 |
| Limitations | 25 | Discuss limitations at study and outcome level (e.g., risk of bias), and at review-level (e.g., incomplete retrieval of identified research, reporting bias). | 9 |
| Conclusions | 26 | Provide a general interpretation of the results in the context of other evidence, and implications for future research. | 9 |
| **FUNDING** | | |  |
| Funding | 27 | Describe sources of funding for the systematic review and other support (e.g., supply of data); role of funders for the systematic review. | 9 |

**Supplementary Table 2**

Revised Cochrane risk-of-bias tool for randomized trials (RoB 2)

**Version of 22 August 2019**

| **Study details**   \| **Reference** \|  \| \| --- \| --- \|   **Study design**   \| X \| Individually-randomized parallel-group trial \| \| --- \| --- \| \| □ \| Cluster-randomized parallel-group trial \| \| □ \| Individually randomized cross-over (or other matched) trial \|   **For the purposes of this assessment, the interventions being compared are defined as**   \| Experimental: \| Infra-popliteal Drug- eluting stenting \| Comparator: \| Standard balloon angioplasty with bailout bare metal stenting \| \| --- \| --- \| --- \| --- \|  \| **Specify which outcome is being assessed for risk of bias** \| Primary Patency \| \| --- \| --- \|  \| **Specify the numerical result being assessed.** \| HR 2.17, 95% CI 1.58-2.97, P 0.00001 \| \| --- \| --- \|   **Is the review team’s aim for this result…?**   \| X \| to assess the effect of *assignment to intervention* (the ‘intention-to-treat’ effect) \| \| --- \| --- \| \| □ \| to assess the effect of *adhering to intervention* (the ‘per-protocol’ effect) \|   **Which of the following sources were obtained to help inform the risk-of-bias assessment? (tick as many as apply)**  X Journal article(s) with results of the trial  X Trial protocol  X Statistical analysis plan (SAP)  □ Non-commercial trial registry record (e.g. ClinicalTrials.gov record)  □ Company-owned trial registry record (e.g. GSK Clinical Study Register record)  □ “Grey literature” (e.g. unpublished thesis)  □ Conference abstract(s) about the trial  □ Regulatory document (e.g. Clinical Study Report, Drug Approval Package)  □ Research ethics application  □ Grant database summary (e.g. NIH RePORTER or Research Councils UK Gateway to Research)  □ Personal communication with trialist  □ Personal communication with the sponsor |
| --- | --- | --- | --- | --- | --- | --- | --- | --- | --- | --- | --- | --- | --- | --- | --- | --- | --- | --- | --- | --- |

**Domain 1: Risk of bias arising from the randomization process**

| **Signalling questions** | **Bosiers, 2012** | **Falkowski, 2009** | **Karnabatidis, 2011** | **Rastan, 2011** | **Scheinert, 2012** | **Siablis, 2007** | **Siablis, 2014** | **Spreen, 2016** | **Tepe, 2010** |
| --- | --- | --- | --- | --- | --- | --- | --- | --- | --- |
| **1.1 Was the allocation sequence random?** | Y | Y | N | Y | Y | N | Y | Y | Y |
| **1.2 Was the allocation sequence concealed until participants were enrolled and assigned to interventions?** | PY | Y | NI | Y | PY | NI | Y | Y | Y |
| **1.3 Did baseline differences between intervention groups suggest a problem with the randomization process?** | N | N | NI | N | N | N | N | N | Y |
| **Risk-of-bias judgement** | **LOW** | **LOW** | **SOME CONCERNS** | **LOW** | **LOW** | **SOME CONCERNS** | **LOW** | **LOW** | **SOME CONCERNS** |
| Optional: What is the predicted direction of bias arising from the randomization process? |  |  |  |  |  |  |  |  |  |

*Y- yes, N- no, PY- probably yes, PN- probably no, NI- no information.*

Domain 2: Risk of bias due to deviations from the intended interventions (*effect of assignment to intervention*)

| **Signalling questions** | **Bosiers, 2012** | **Falkowski, 2009** | **Karnabatidis, 2011** | **Rastan, 2011** | **Scheinert, 2012** | **Siablis, 2007** | **Siablis, 2014** | **Spreen, 2016** | **Tepe, 2010** |
| --- | --- | --- | --- | --- | --- | --- | --- | --- | --- |
| **2.1. Were participants aware of their assigned intervention during the trial?** | N | PN | PY | N | PN | PY | PN | PY | PN |
| **2.2. Were carers and people delivering the interventions aware of participants' assigned intervention during the trial?** | Y | Y | Y | N | Y | Y | Y | Y | Y |
| **2.3. If Y/PY/NI to 2.1 or 2.2: Were there deviations from the intended intervention that arose because of the trial context?** | N | N | N | - | N | N | N | N | N |
| **2.4 If Y/PY to 2.3: Were these deviations likely to have affected the outcome?** | - | - | - | - |  | - | - | - | - |
| **2.5. If Y/PY/NI to 2.4: Were these deviations from intended intervention balanced between groups?** | - | - | - | - |  | - | - | - | - |
| **2.6 Was an appropriate analysis used to estimate the effect of assignment to intervention?** | Y | PY | Y | Y | Y | Y | Y | Y | NI |
| **2.7 If N/PN/NI to 2.6: Was there potential for a substantial impact (on the result) of the failure to analyse participants in the group to which they were randomized?** | - | N | - | - |  | - | - | - | PN |
| **Risk-of-bias judgement** | LOW | LOW | LOW | LOW | LOW | LOW | LOW | LOW | SOME CONERNCS |
| Optional: What is the predicted direction of bias due to deviations from intended interventions? |  |  |  |  |  |  |  |  |  |

Domain 2: Risk of bias due to deviations from the intended interventions (*effect of adhering to intervention*)

| **Signalling questions** | **Bosiers, 2012** | **Falkowski, 2009** | **Karnabatidis, 2011** | **Rastan, 2011** | **Scheinert, 2012** | **Siablis, 2007** | **Siablis, 2014** | **Spreen, 2016** | **Tepe, 2010** |
| --- | --- | --- | --- | --- | --- | --- | --- | --- | --- |
| **2.1. Were participants aware of their assigned intervention during the trial?** | N | PN | PY | N | PN | PY | N | Y | PN |
| **2.2. Were carers and people delivering the interventions aware of participants' assigned intervention during the trial?** | Y | Y | PY | N | Y | PY | Y | Y | Y |
| **2.3. [If applicable:] If Y/PY/NI to 2.1 or 2.2: Were important non-protocol interventions balanced across intervention groups?** | PY | Y | N | - | PY | N | PY | N | Y |
| **2.4. [If applicable:] Were there failures in implementing the intervention that could have affected the outcome?** | N | N | N | N | N | N | N | N | N |
| **2.5. [If applicable:] Was there non-adherence to the assigned intervention regimen that could have affected participants’ outcomes?** | N | N | N | N | N | N | N | N | N |
| **2.6. If N/PN/NI to 2.3, or Y/PY/NI to 2.4 or 2.5: Was an appropriate analysis used to estimate the effect of adhering to the intervention?** | - | - | Y | - | - | Y | - | PY | - |
| **Risk-of-bias judgement** | LOW | LOW | SOME CONCERNS | LOW | LOW | SOME CONCERNS | LOW | SOME CONCERNS | LOW |
| Optional: What is the predicted direction of bias due to deviations from intended interventions? |  |  |  |  |  |  |  |  |  |

Domain 3: Missing outcome data **(outcome in this case is patency)**

| **Signalling questions** | **Bosiers, 2012** | **Falkowski, 2009** | **Karnabatidis, 2011** | **Rastan, 2011** | **Scheinert, 2012** | **Siablis, 2007** | **Siablis, 2014** | **Spreen, 2016** | **Tepe, 2010** |
| --- | --- | --- | --- | --- | --- | --- | --- | --- | --- |
| **3.1 Were data for this outcome available for all, or nearly all, participants randomized?** | Y | Y | PY | Y | Y | Y | Y | Y | PY |
| **3.2 If N/PN/NI to 3.1: Is there evidence that the result was not biased by missing outcome data?** | - | - | - | - | - | - | - | - | - |
| **3.3 If N/PN to 3.2: Could missingness in the outcome depend on its true value?** | - | - | - | - | - | - | - | - | - |
| **3.4 If Y/PY/NI to 3.3: Is it likely that missingness in the outcome depended on its true value?** | - | - | - | - | - | - | - | - | - |
| **Risk-of-bias judgement** | LOW | LOW | LOW | LOW | LOW | LOW | LOW | LOW | LOW |
| Optional: What is the predicted direction of bias due to missing outcome data? |  |  |  |  |  |  |  |  |  |

Domain 4: Risk of bias in measurement of the outcome

| **Signalling questions** | **Bosiers, 2012** | **Falkowski, 2009** | **Karnabatidis, 2011** | **Rastan, 2011** | **Scheinert, 2012** | **Siablis, 2007** | **Siablis, 2014** | **Spreen, 2016** | **Tepe, 2010** |
| --- | --- | --- | --- | --- | --- | --- | --- | --- | --- |
| **4.1 Was the method of measuring the outcome inappropriate?** | N | N | N | N | N | N | N | N | N |
| **4.2 Could measurement or ascertainment of the outcome have differed between intervention groups?** | N | N | N | N | N | N | N | N | N |
| **4.3 If N/PN/NI to 4.1 and 4.2: Were outcome assessors aware of the intervention received by study participants?** | PN | NI | PN | PN | PN | N | PY | PY | PN |
| **4.4 If Y/PY/NI to 4.3: Could assessment of the outcome have been influenced by knowledge of intervention received?** | - | Y | - | - | - | - | Y | Y | - |
| **4.5 If Y/PY/NI to 4.4:** **Is it likely that assessment of the outcome was influenced by knowledge of intervention received?** | - | PN | - | - | - | - | PN | N | - |
| **Risk-of-bias judgement** | LOW | SOME CONCERNS | LOW | LOW | LOW | LOW | SOME CONCERNS | SOME CONCERNS | LOW |
| Optional: What is the predicted direction of bias in measurement of the outcome? |  |  |  |  |  |  |  |  |  |

Domain 5: Risk of bias in selection of the reported result

| **Signalling questions** | **Bosiers, 2012** | **Falkowski, 2009** | **Karnabatidis, 2011** | **Rastan, 2011** | **Scheinert, 2012** | **Siablis, 2007** | **Siablis, 2014** | **Spreen, 2016** | **Tepe, 2010** |
| --- | --- | --- | --- | --- | --- | --- | --- | --- | --- |
| **5.1 Were the data that produced this result analysed in accordance with a pre-specified analysis plan that was finalized before unblinded outcome data were available for analysis?** | PY | PY | Y | Y | Y | PY | Y | PY | Y |
| **Is the numerical result being assessed likely to have been selected, on the basis of the results, from...** |  |  |  |  |  |  |  |  |  |
| **5.2. ... multiple eligible outcome measurements (e.g. scales, definitions, time points) within the outcome domain?** | N | N | N | N | N | N | N | Y | N |
| **5.3 ... multiple eligible analyses of the data?** | N | N | N | N | N | N | N | N | N |
| **Risk-of-bias judgement** | LOW | LOW | LOW | LOW | LOW | LOW | LOW | HIGH | LOW |
| Optional: What is the predicted direction of bias due to selection of the reported result? |  |  |  |  |  |  |  |  |  |

**Overall risk of bias**

| **Risk-of-bias judgement** | **Bosiers, 2012** | **Falkowski, 2009** | **Karnabatidis, 2011** | **Rastan, 2011** | **Scheinert, 2012** | **Siablis, 2007** | **Siablis, 2014** | **Spreen, 2016** | **Tepe, 2010** |
| --- | --- | --- | --- | --- | --- | --- | --- | --- | --- |
|  | LOW | SOME CONCERNS | SOME CONCERNS | LOW | LOW | SOME CONCERNS | SOME CONCERNS | SOME CONCERNS | SOME CONCERNS |

**SUPPLEMENTARY FIGURES**

**Supplementary Figure 1:** Primary patency in infrapopliteal disease among DES vs control in patients with critical limb ischemia.

**Supplementary Figure 2:** Target lesion revascularization in infrapopliteal disease among DES vs control in patients with critical limb ischemia.

**Supplementary Figure 3:** Major amputations at maximum follow-up among DES vs control in patients with critical limb ischemia.

**Supplementary Figure 4:** All-cause mortality occurrence among DES vs control in patients with critical limb ischemia.

**
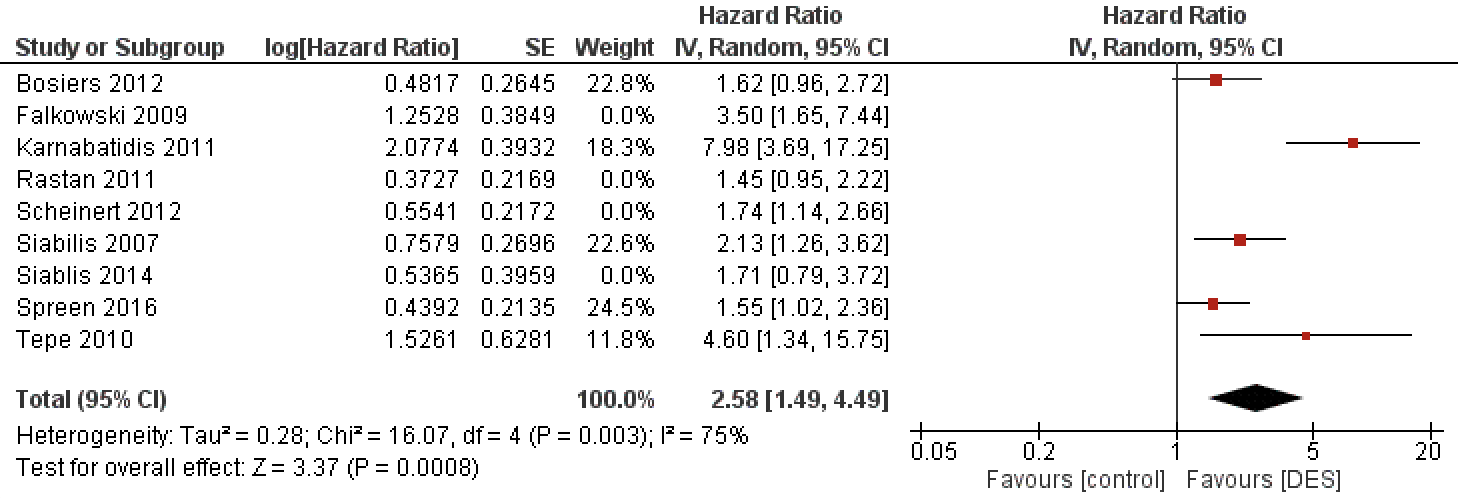
**

**Supplementary Figure 1**: Primary patency in infrapopliteal disease among DES vs control in patients with critical limb ischemia.


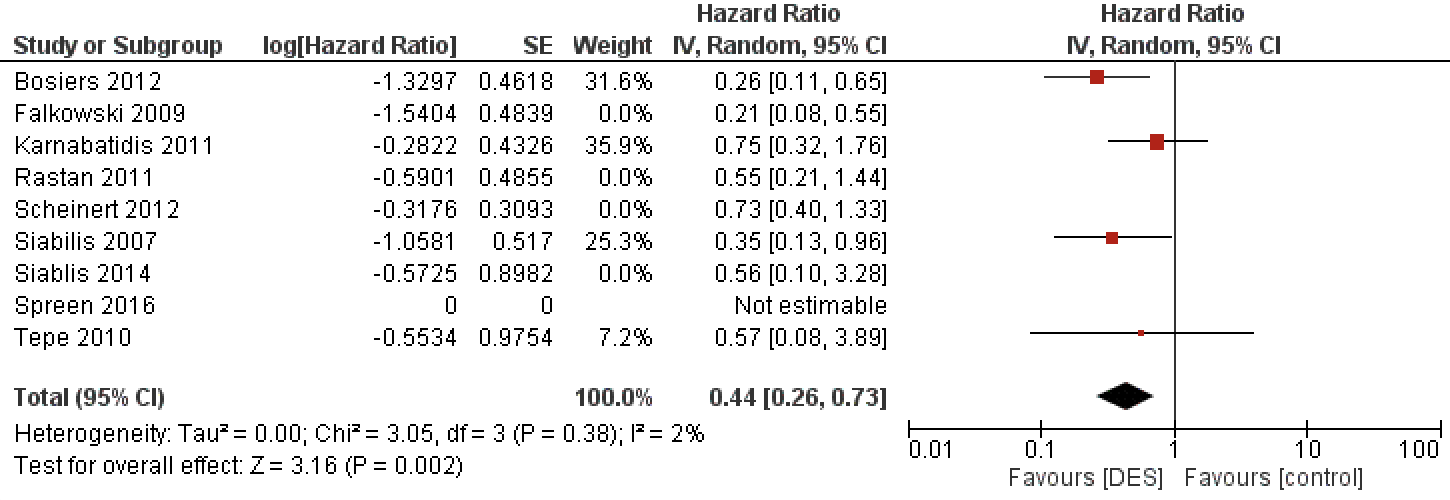


**Supplementary Figure 2**: Target lesion reintervention in infrapopliteal disease among DES vs control in patients with critical limb ischemia.


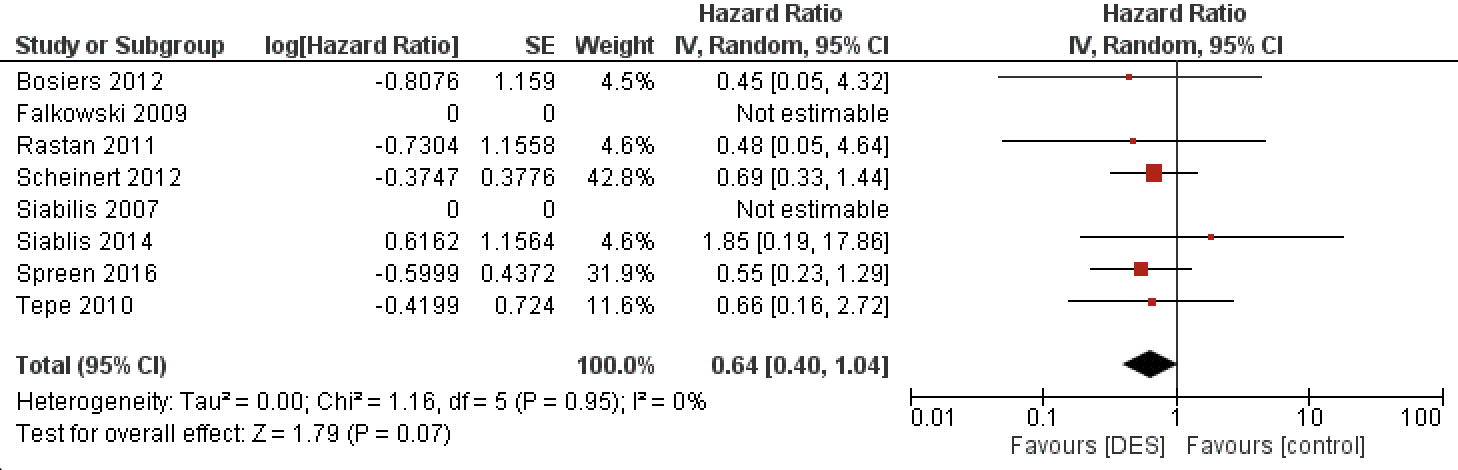


**Supplementary Figure 3**: Major amputations at maximum follow-up among DES vs control in patients with critical limb ischemia.

**
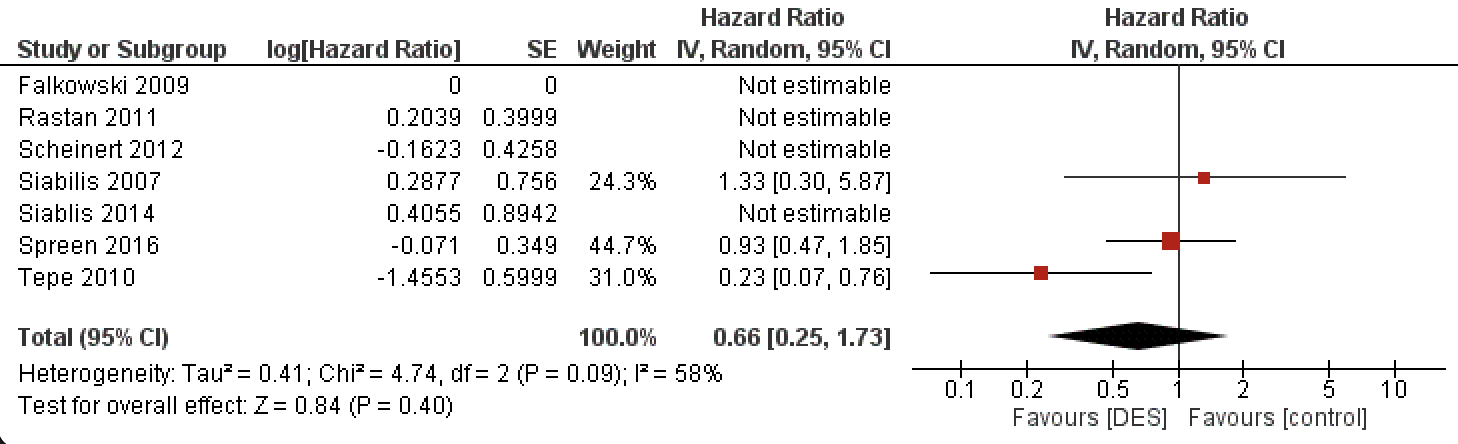
**

**Supplementary Figure 4**: All-cause mortality occurrence among DES vs control in patients with critical limb ischemia.
